# SynerGNet: A Graph Neural Network Model to Predict Anticancer Drug Synergy

**DOI:** 10.3390/biom14030253

**Published:** 2024-02-21

**Authors:** Mengmeng Liu, Gopal Srivastava, J. Ramanujam, Michal Brylinski

**Affiliations:** 1 Division of Electrical and Computer Engineering, Louisiana State University, Baton Rouge, LA 70803, USA; lmengm1@lsu.edu (M.L.);; 2 Department of Biological Sciences, Louisiana State University, Baton Rouge, LA 70803, USA; 3 Center for Computation and Technology, Louisiana State University, Baton Rouge, LA 70803, USA

**Keywords:** drug combination, cancer treatment, graph neural network, drug synergistic effects, drug antagonistic effects, data augmentation

## Abstract

Drug combination therapy shows promise in cancer treatment by addressing drug resistance, reducing toxicity, and enhancing therapeutic efficacy. However, the intricate and dynamic nature of biological systems makes identifying potential synergistic drugs a costly and time-consuming endeavor. To facilitate the development of combination therapy, techniques employing artificial intelligence have emerged as a transformative solution, providing a sophisticated avenue for advancing existing therapeutic approaches. In this study, we developed SynerGNet, a graph neural network model designed to accurately predict the synergistic effect of drug pairs against cancer cell lines. SynerGNet utilizes cancer-specific featured graphs created by integrating heterogeneous biological features into the human protein–protein interaction network, followed by a reduction process to enhance topological diversity. Leveraging synergy data provided by AZ-DREAM Challenges, the model yields a balanced accuracy of 0.68, significantly outperforming traditional machine learning. Encouragingly, augmenting the training data with carefully constructed synthetic instances improved the balanced accuracy of SynerGNet to 0.73. Finally, the results of an independent validation conducted against DrugCombDB demonstrated that it exhibits a strong performance when applied to unseen data. SynerGNet shows a great potential in detecting drug synergy, positioning itself as a valuable tool that could contribute to the advancement of combination therapy for cancer treatment.

## 1. Introduction

The current understanding of cancer cellular mechanisms highlights the sophisticated role of various molecular pathways regulating cell growth, division, and survival. Cancer is characterized by abnormalities in signaling networks, often involving genetic mutations, epigenetic alterations, and the dysregulation of key cellular processes [1]. Anticancer drugs play a key role in targeting specific components of these aberrant pathways, aiming to impede or eliminate the uncontrolled proliferation of cancer cells. Chemotherapy [2], targeted therapies [3], and immunotherapies [4] are among the diverse classes of anticancer treatments employed to disrupt the viability of cancer cells. Despite significant advancements in cancer research, the complexity of cellular mechanisms and the heterogeneous nature of tumors pose substantial challenges in comprehensively understanding the disease. The vast molecular diversity among cancer types and even within individual tumors demands a fine-tuned approach to devise innovative treatment strategies, considering the dynamic nature of cancer cells and their ability to adapt to therapeutic interventions.

Analyzing the mechanisms underlying cancer and the interactions between anticancer drugs and cancer cells is a multifaceted task fraught with challenges. The heterogeneity of cancer cells within a tumor and across different patients makes it challenging to develop universally effective treatments. Additionally, the intricate signaling pathways and feedback loops within cellular networks contribute to the dynamic and adaptive nature of cancer cells, often leading to resistance against therapeutic interventions [5]. Drug combination therapy emerged as a promising strategy to tackle these issues [6,7,8]. The concurrent use of multiple anticancer drugs often surpasses the efficacy of monotherapies by synergistically or additively modulating distinct critical pathways. Combining drugs with diverse mechanisms of action not only enhances therapeutic efficacy but also facilitates the use of lower doses, thereby reducing the potential for side effects and toxicity [6,9,10]. Despite its potential benefits, the pursuit of effective drug combinations encounters numerous challenges due to the large number of potential drug pairs, leading to a combinatorial explosion [11]. Consequently, identifying synergistic combinations through experimental methods alone is a highly costly and time-consuming endeavor, even when employing high-throughput screening. To expedite the development of combination therapy, artificial intelligence (AI) techniques have gained popularity as an efficient computational method for predicting novel and effective drug combinations [12].

Current AI techniques to predict drug combinations can be classified into two broad categories: traditional machine learning (ML) and deep learning (DL) methods. Most widely adopted ML approaches include decision trees (DTs) [13], random forest (RF) [14], support vector machines (SVMs) [15], factorization machines (FMs) [16], logistic regression (LR) [17], naïve Bayes (NB) classifiers [18], and extreme gradient boosting (XGBoost) [19]. These methods typically excel for small and medium datasets but often encounter difficulties in fully leveraging the potential of large datasets. Due to their relatively low prediction accuracy, single ML models are mostly used as baselines. Studies adopting ML algorithms as analyzing tools often integrate a series of classifiers or regressors through ensemble learning [12]. For example, the top winning method in the AstraZeneca Dialog for Reverse Engineering Assessments and Methods (AZ-DREAM) Challenges was developed based on a certain number of RFs. Each tree in the forest was built from the most predictive feature to the next predictive one, until it reached a node that was sufficiently small. Then, they were resampled numerous times and the aggerated results were utilized as the final output of the model [20]. Further, a two-layer multiple classifier system (TLMCS) was developed by combining multiple SVMs [21]. In the first layer, five SVMs were individually trained against distinct types of features. Subsequently, the outputs of these five base predictors were combined into a vector serving as an input to the SVM in the second layer. Another model to predict drug pairs demonstrating species-selective toxicity against human fungal pathogens is second order naïve Bayesian and random forest (SONAR) [22]. First a NB model was constructed to predict genetic sensitivities based on structural features and chemical–genetic interaction matrix (CGM) data. Then, the feature set derived from CGM and the predicted likelihood scores from the NB model were utilized as input for the RF model.

The availability of large-scale drug combination data facilitates the training of deep neural network architectures. Compared to traditional ML methods, DL approaches have better capabilities to learn complex and nuanced patterns, capturing the intricate relationships among cellular, molecular, and biological system-level features. DeepSynergy [23], one of the first deep learning methods to predict drug combinations, showed obvious superiority over gradient boosting machines, RFs, SVMs, and elastic nets in predicting the synergy of anticancer drugs. DeepSynergy is a multi-layer feed-forward neural network, which integrates chemical and genomic information as input information, and utilizes normalization strategies to address the heterogeneity of input data. However, this model may not generalize well to unseen data where novel drugs or cell lines are involved. Deep tensor factorization (DTF) [24] combines a fully connected neural network with a tensor factorization method to predict anticancer drug synergy. The weighted tensor factorization is used to extract features from drug synergy data. The extracted latent features are then fed into the network to predict the drug synergy status. The performance of DTF is comparable to that of DeepSynergy. Further, AuDNNsynergy [25] incorporates three autoencoders, each equipped with fully connected layers, to predict the synergy scores of drug combinations. The model utilizes one autoencoder to obtain each of the following feature representations: gene expression, copy number variation, and mutation of cancer cell lines. Subsequently, the encoded features are combined with the physicochemical features of individual drugs as the input of fully connected layers to predict the synergy values for given drug pairs.

The abovementioned DL models require data to be in the Euclidean representation format, characterized by a regular, fixed-size structure. However, the intrinsic heterogeneity and complexity of biological networks render traditional Euclidean representations inadequate for mapping and reflecting the diverse properties of biological information. In contrast, graph-structured data prove highly effective in capturing relationships among various biological entities, such as genes, proteins, ligands, metabolites, drugs, diseases, or even medical database records [25]. Consequently, graph neural networks (GNNs), an efficient and promising deep learning technique in the non-Euclidean space, have become a powerful tool for advancing the comprehension of biological systems and accelerating discoveries of synergistic drugs [11,26]. For instance, a novel GNN model was developed to predict cell line specific synergistic drug combinations [27]. This approach utilizes multimodal graphs for various cell lines created by integrating the drug–drug combination, drug–protein interaction, and protein–protein interaction (PPI) networks. The GNN model comprises an encoder featuring four graph convolution layers and a matrix decoder. In its operation, the encoder takes each graph as an input, producing node-level vector embeddings, while the decoder decodes these embeddings to predict a synergy score for each drug combination. Notably, the model performance, particularly in terms of the area under the receiver operating characteristic (ROC), closely aligns with that of DeepSynergy. It has been pointed out that limitations in available drug–protein interaction data contribute to certain associations remaining undiscovered within cell line specific networks.

The graph autoencoder with the convolutional neural network for drug synergy prediction (GAECDS) employs a two-layer graph convolution module as an encoder to embed drug–drug synergy features and a multilayer perceptron module to embed cell features [28]. Subsequently, the generated features are combined and fed into a convolutional neural network to predict drug synergy. Another recent approach, AttenSyn, utilizes an attention-based GNN architecture to identify synergistic drug combinations [29]. The process begins with the conversion of the simplified molecular-input line-entry system (SMILES) profiles of paired drugs into molecular graphs, followed by the integration of cell line features into nodes within these molecular graphs. The generated graphs are processed through a drug-embedding module comprising multiple GNN and LSTM layers designed to yield embeddings for each node within the graphs. An attention-based pooling module is employed to capture the graph-level features followed by a prediction module to combine drug embeddings with cell line features and predict synergistic drug combinations.

Notwithstanding these encouraging reports, there are some shortcomings in the existing methods. First, even with the existence of extensive synergy databases, there is a limited diversity in terms of cell lines, drugs, and drug combinations [27]. As a result, only a limited number of combinations can be investigated by cell line specific approaches. Second, despite employing graph structures to represent biological networks, existing methods usually process the drug information and cell line features independently and then combine them in an additive manner [28]. This approach might not adequately capture the complex biological relationships existing between molecular and cellular entities. Moreover, numerous studies employ SMILES as drug features in model training, rather than exploiting more fundamental interactions between drugs and proteins [29,30,31]. Although models trained on such data may exhibit high prediction accuracy, they lack generalizability to unseen data, as they tend to memorize existing data rather than capture the underlying patterns of drug–cell interactions.

To address these issues, we propose SynerGNet, a deep GNN-based model to predict the synergistic effect of drug combinations. SynerGNet utilizes generalized graph convolution (GENConv) architecture to construct graph convolutional modules and incorporates strong regularization mechanisms. The input to SynerGNet is a featured graph that directly integrates heterogeneous molecular and cellular features into the human PPI network. The featured graphs and GENConv-based convolutional modules enable SynerGNet to learn the intricate biological relationships between drugs and cell lines, instead of just memorizing drug representations. SynerGNet is first trained on the AZ-DREAM Challenges dataset and compared to both traditional machine learning and deep learning methods. Next, all drug synergy classifiers are re-trained against a large-scale augmented data derived from the AZ-DREAM Challenges database to eliminate the issue of a restricted variety of cell lines, drugs, and drug combinations. Finally, we conduct an independent validation of deep learning techniques against unseen data comprising drug molecules with a very low chemical similarity to the training compounds. Overall, SynerGNet not only demonstrates considerable potential in the precise detection of drug synergy, but also positions itself as a valuable tool that could contribute to the advancement of combination cancer therapy. Its robust performance offers promising avenues for advancing the effectiveness of therapeutic approaches in the complex landscape of cancer treatment.

## 2. Materials and Methods

### 2.1. Protein–Protein Interaction Network

The human PPI network was constructed using IHP-PING [32] that integrates interaction data from multiple databases, including StringDB [33], BioGRID [34], DIP [35], HPRD [36], IntAct [37], MINT [38], MPPI-MIPS [39], with functional information from UniProt [40]. Applying a threshold of ≥0.7, indicating a high level of confidence in the data [32], resulted in a PPI network comprising 18,997 nodes. Based on the drug target data from AZ-DREAM Challenges [20], 14,374 nodes are categorized as druggable and 4623 as non-druggable.

### 2.2. Node Features

#### 2.2.1. Genomic Alterations

Differential gene expression data were extracted from the cancer cell line encyclopedia (CCLE) [41], a comprehensive repository providing extensive genetic characterizations for various cancer cell lines. Specifically, we obtained the differential gene expression information for 16,225 genes in the PPI network and a subset of 82 cell lines from AZ-DREAM Challenges. The expression values were encoded as 0 (normally expressed), +1 (up-regulated), and −1 (down-regulated). Gene copy number variation (CNV) and mutation data were retrieved from AZ-DREAM Challenges. CNV data in terms of wild-type, amplification, and deletion are available for 494 genes across 82 cancer cell lines. The mutation data encompass 14,169 genes and the following 13 distinct mutation types: complex compound substitution, complex in-frame deletion, complex frameshift, complex in-frame insertion, frameshift deletion, in-frame deletion, frameshift insertion, in-frame insertion, nonstop extension, silent coding substitution, missense substitution, nonsense substitution, and unknown.

#### 2.2.2. Gene Ontology Terms

Gene ontology (GO) terms for all 18,997 genes within the human PPI network were retrieved from the GO database [42]. Utilizing ANC2Vec [43], a total of 12,661 GO terms across the dataset were transformed into 200-dimensional vectors. Subsequently, each node in the network was assigned a summation vector derived from the embeddings of individual GO terms associated with the corresponding gene.

#### 2.2.3. Drug–Protein Associations

Scores indicating drug–protein associations were extracted from the search tool for interactions of chemicals (STITCH) database [44]. Associations in this database are assigned scores ranging from 150 to 999 representing the confidence level of the interaction between a specific drug and protein, with higher scores indicating a greater interaction confidence.

### 2.3. Graph Statistics

Full-size and reduced graphs were analyzed in terms of their average node degree, graph density, and graph diameter. The average node degree $\bar{k}$ is the average number of connections per node calculated as:

(1) $$\bar{k}=\frac{2\left[E\right]}{\left[V\right]}$$

where $\left[E\right]$ is the total number of edges and $\left[V\right]$ is the total number of nodes in the graph. The density of a graph is determined by dividing the number of edges by the maximum possible number of edges between nodes. For an undirected graph without self-loops, the density ρ is:

(2) $$\rho =\frac{2\left[E\right]}{\left[V\right]\left(\left[V\right]-1\right)}$$

The concept of density gauges the “fullness” of a graph. A low-density value signifies a sparse graph, indicating that it has fewer connections, while a high-density value suggests a dense graph with a greater number of connections. Finally, the graph diameter D is defined as the maximum length of the shortest path between any two nodes in the graph:

(3) $$D=max_{u.v\in V\left(G\right)}d\left(u,v\right)$$

where $V\left(G\right)$ is the set of nodes in the graph G, and $d\left(u,v\right)$ represents the shortest path length between nodes u and v.

### 2.4. Drug Action/Chemical Similarity Score

In this study, the data augmentation protocol utilizes the drug action/chemical similarity (DACS) metric to choose candidate drugs for substituting each compound in the original combination [45]. The DACS score computation involves assessing drugs’ structural and molecular target similarities. Structural similarity is calculated using the FP2 fingerprint-based Tanimoto index against the STITCH database [44], with a Tanimoto cutoff of ≤0.95 applied to ensure structural diversity in the selection of molecules. This choice is supported by the fact that the TC range of (0.95, 1) corresponds to full identity in terms of molecular similarity [46]. Molecular target similarity is computed as the Matthews correlation coefficient against 18,997 nodes in the PPI network [32]. Drug candidates for augmentation were selected at a DACS cutoff of ≥0.53, which ensures that the majority of substitute drugs exhibit similar pharmacological profiles to their parent molecules [45]. Subsequently, new drug combinations were created by pairing the selected candidate molecules with the other drug from the original pair in the AZ-DREAM Challenges synergy dataset.

### 2.5. Graph Data Vectorization

In order to employ the RF classifier, cancer-specific featured graphs were transformed into fixed-size vectors. First, nodes within each graph were sorted by their node degree in descending order to ensure a consistent node sequence across graphs, minimizing potential biases induced by node order. Subsequently, the features of each node were standardized by removing the mean and scaling to unit variance:

(4) $$\hat{x}=\frac{x-\mu_{x}}{\delta_{x}}$$

where $\hat{x}$ is the standardized feature value, x is the original feature value, and $\mu_{x}$ is the mean of the feature values with the corresponding standard deviation $\delta_{x}$. After standardization, the principal component analysis (PCA) [47,48] was applied to the resulting 218-dimensional feature vectors, reducing the feature space to 32 principal components, capturing the most significant variance within the data. In the final step, the feature matrix of each graph was flattened to a single vector, resulting in vectors with an average dimension of 43,904 (the number of features per node × the average number of nodes per graph). The vectorized graphs were then utilized to train RF models.

### 2.6. Training and Validation

The performance of RF and SynerGNet classifiers was evaluated using a 5-fold cross-validation, a widely recognized and accepted method in machine learning and statistical analysis for model validation. The original set of 3272 instances were divided into five equal subsets. Within each fold, one subset of the data was allocated as the test set while the remaining subsets constituted the training set. One of the key aspects of this study was to examine the impact of integrating augmented data during the training phase by supplementing each training set with synthetic data generated based on the corresponding original training instances. This strategy was crucial to prevent data leakage, ensuring that no augmented data originating from the test instances were included in the training set. It is important to note that each testing set exclusively comprised the original drug combinations with experimentally established synergy scores.

To validate the predictive power of SynerGNet, we conducted an independent validation against the DrugCombDB [49] dataset. This external dataset provided an unbiased platform to properly evaluate the generalizability of machine learning models. During this independent validation, SynerGNet underwent training with two distinct settings: one model was trained solely using the original AZ-DREAM Challenges dataset, while another model was trained using a combination of the original and augmented datasets. In both settings, the SynerGNet model was trained for 200 epochs. Following training, each model iteration was tested on the DrugComb data to assess the impact of training with augmented data on the model performance.

### 2.7. Evaluation Metrics

The receiver operating characteristic (ROC) curve is a graphical representation of a model’s ability to distinguish between two classes, typically positive and negative, across different threshold values. The area under the ROC curve (AUC), is a metric used to evaluate the performance of a classification model, particularly in binary classification problems. The AUC is a single value that quantifies the overall performance of a model based on the ROC curve. A perfect model has an AUC of 1.0, indicating the perfect discrimination between positive and negative classes. A random or ineffective model has an AUC of 0.5, which is equivalent to the area under the diagonal line (no discrimination).

Balanced accuracy (BAC) is another metric used to evaluate the performance of a classification model, particularly when the distribution of classes is imbalanced. It takes into account both the sensitivity (true positive rate) and specificity (true negative rate) of a model, providing a more balanced assessment across different classes. BAC values range from 0 to 1, with higher values indicating a better overall performance. A BAC of 0.5 suggests a random chance, while a BAC of 1.0 indicates a perfect classification.

The positive predictive value (PPV), also known as the precision, is a metric used to evaluate the accuracy of positive predictions made by a classification model. It assesses the proportion of predicted positive instances that are true positives. Precision is particularly important in situations where the consequences of predicting false positives are highly detrimental. A precision value of 1.0 indicates perfect precision, where all positive predictions made by the model are correct. A lower precision value suggests that there are false positives in the predictions.

The false positive rate (FPR) is a metric used to assess the proportion of negative instances that are incorrectly classified as positive by a classification model. It is calculated as the ratio of false positives to the sum of false positives and true negatives. The FPR is complementary to specificity and is particularly relevant when the cost or consequences of false positives are a concern. A lower FPR value indicates that the model has a lower tendency to incorrectly classify negative instances as positive, whereas a higher FPR suggests that the model has a higher rate of false positives.

The Matthews correlation coefficient (MCC) is a metric used to evaluate the performance of binary classification models, particularly when dealing with imbalanced datasets. It takes into account true positives, true negatives, false positives, and false negatives to provide a balanced measure of the classification performance. High MCC values indicate a better overall performance.

## 3. Results

### 3.1. Cancer-Specific Featured Graphs

**Figure 1:**
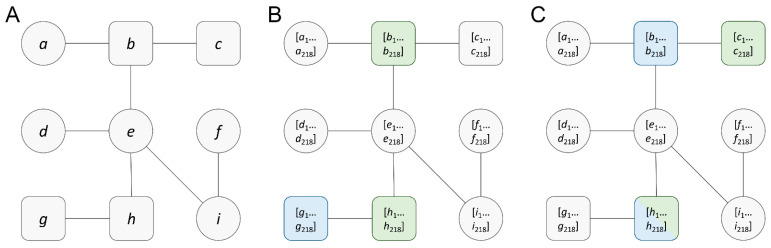
Construction of full-size featured graphs. (**A**) A simplified human PPI network, serving as a base graph before mapping any features onto it. Round squares represent druggable nodes (*b*, *c*, *g*, *h*) while circles represent non-druggable nodes (*a*, *d*, *e*, *f*, *i*). (**B**,**C**) Featured graphs for two different drug pairs and cancer cell lines. These graphs have the same topology and druggable/non-druggable nodes as the base graph. Each node is embedded with a 218-dimensional feature vector, which is composed of gene expression (1 value), gene copy number variation (3 values), gene mutation (13 values), affinity score (1 value), and gene ontology terms (200 values). Green squares are target nodes for one drug and blue squares for the second drug. Both drugs in a pair can share a target node, colored green/blue in (**C**).

To explore the complex relationships between cancer cellular mechanisms and the biochemical interactions of anticancer drugs, we employ a graph structure to model cancer-specific biological networks. Figure 1 shows that these graphs are constructed by mapping differential gene expression, gene copy number variation, gene mutation, gene ontology terms, and drug–protein associations into the human PPI network. The PPI network shown in Figure 1A, consisting of druggable (rounded squares) and non-druggable (circles) nodes, serves as a base graph. For a given pair of drugs and a cell line, each protein in the PPI network is assigned a vector of 218 features (Figure 1B,C). Through this graph construction procedure, anticancer drug synergy instances consisting of paired drugs and their target cancer cell line are converted into featured graphs.

Despite effectively mapping complex biological relationships, cancer-specific graphs pose certain challenges in terms of data representation within the machine learning framework. First, featured graphs constructed for different drug pairs, such as those shown in Figure 1B,C, have the same graph topology, and the differences only lie in node features that depend on both drugs and the cell line. The consistent presence of identical graph topologies across multiple instances poses a significant challenge for a GNN model to extract the necessary information for effective learning. Second, according to the graph statistics reported in Table 1, each full-size graph contains 18,997 nodes (proteins) and 697,185 edges (interactions between proteins) with an average node degree of 73.40. Training a GNN model on a graph of this size can be computationally intensive, because GNNs require the computation of node embeddings, which frequently involves aggregating features from neighboring nodes. Consequently, with an increase in the average node degree, the computational requirements for each node increase during each propagation step. In the context of GNNs, the storage of intermediate node embeddings across layers can become memory-intensive. This challenge is particularly pronounced in large graphs, especially when deploying deep GNN architectures on systems with a limited memory capacity. Third, the full-size graph diameter of nine further increases the computational complexity and deepens the network required to capture long-range dependencies, which may lead to over-smoothing and optimization challenges.

### 3.2. Graph Reduction to Create Topological Diversity

**Figure 2:**
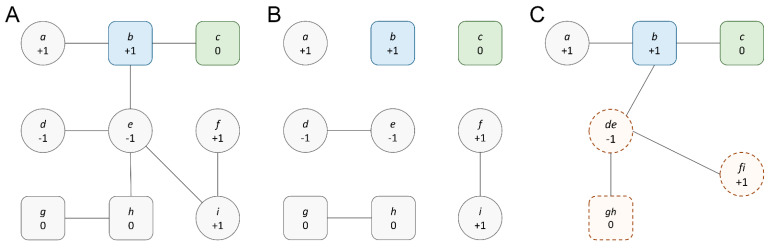
Graph reduction through edge contraction. (**A**) A full-size graph comprising druggable nodes (round squares: *b*, *c*, *g*, *h*) and non-druggable nodes (circles: *a*, *d*, *e*, *f*, *i*). A green square represents the target node. Differential gene expression is denoted by 0 (normally expressed), +1 (up-regulated), and −1 (down-regulated). (**B**) An intermediate graph constructed by removing edges connecting the target node, druggable and non-druggable nodes, and those nodes having dissimilar differential gene expression. Remaining edges are going to be contracted to produce merged nodes. (**C**) A reduced graph with merged druggable nodes shown as round red squares with a dashed outline (*de*, *fi*) and merged non-druggable nodes represented by red circles with a dashed outline (*gh*). Those edges previously removed from the full-size graph are put back to restore the original graph connectivity.

To address the abovementioned issues with full-size graphs, we devised a knowledge-based graph reduction procedure through edge contraction, a fundamental operation in graph theory [50,51]. The idea is to merge nodes that are similar in both their potential interaction with drugs and their activity levels (Figure 2). The potential interaction of a protein with drugs is indicated through drug–protein association by identifying whether a protein is a drug target protein. Protein activity levels are measured by their gene expression because proteins with the same gene expression can have similar functional states or activities. First, from an initial full-size graph (Figure 2A), edges connecting druggable (proteins known to be targeted by drugs) and non-druggable (proteins not targeted by drugs) nodes and those connecting target nodes are removed. This procedure ensures that proteins having different interactions with drugs are not going to be merged. Further, target nodes become isolated because these proteins are essential to preserve the distinct drug–protein interactions within each specific instance to ensure that the analysis of individual drug pair effects is accurate. Subsequently, edges connecting proteins having different gene expression levels are also removed, so that merged nodes have the same activity levels. After this step, only nodes with similar drug interactions and the same gene expression are still connected (Figure 2B). Finally, the persisting edges are contracted to produce merged nodes. The feature vectors of merged nodes are updated and all the previously removed edges are restored, resulting in the final reduced cancer-specific graph (Figure 2C).

### 3.3. Analysis of Reduced Cancer-Specific Networks

The statistics for reduced graphs are reported in Table 1. A significant reduction in the number of nodes (from 18,997 to an average of 1372) and edges (from 697,185 to an average of 4204) considerably lowers the computational requirements for training a GNN model, resulting in a faster processing time and a lower memory demand. Furthermore, through the consolidation of less informative nodes and edges, the GNNs can prioritize crucial nodes, edges, and subgraphs, allowing for the extraction of more pertinent patterns and structures from the data. The graph diameter decreases from nine to six, indicating that the maximum shortest path between any two nodes in the graph is smaller in reduced graphs compared to the original full-size graphs. This can enhance information propagation in the GNNs by reducing the number of steps required for information to reach distant nodes. Also, a higher density of the reduced graphs can aid in GNN learning, as nodes tend to have richer local information. The average node degree is considerably reduced from 73.4 to around 5.8, which can lead to a more uniform distribution of information and reduce the dominance of high-degree nodes that may surpass nodes with fewer connections in the graph. The initial high ratio of druggable to non-druggable nodes of 3.1 for the full-size graph decreased to 0.29 ± 0.15 for reduced graphs providing a more precise emphasis on the drug targets associated with a specific drug pair. The comparison of various graph properties shows that reduced graphs not only lower computational requirements, but also offer potential advantages in terms of model generalization, interpretation, and the overall efficiency of the GNN learning process.

### 3.4. Data Augmentation

**Figure 3:**
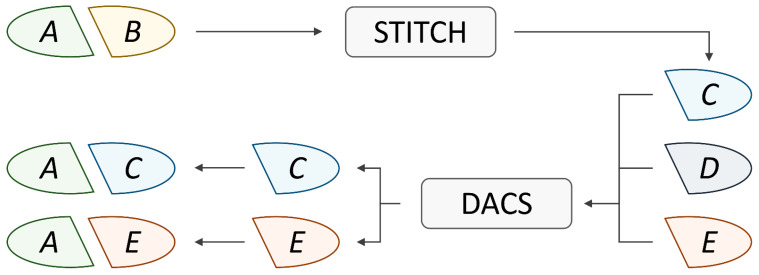
Augmentation procedure to expand drug synergy data. An input is a pair of drugs, *A*:*B* with a known synergy score, one of which will be replaced to generate a set of new instances. Drugs *C*, *D*, and *E* are selected from the STITCH database as candidates to replace drug *B*. DACS similarity scores between drug *B* and drugs *C*, *D*, and *E* are calculated and those molecules above a predefined threshold are retained (*C* and *E*). Drugs *C* and *E* are then used as substitutes for drug *B* creating augmented instances with drug *A*, *A*:*C* and *A*:*E*.

Many drug synergy datasets, such as AZ-DREAM Challenges [20], employ strict criteria for synergy scores to distinctly categorize drug combinations as synergistic or antagonistic. However, this frequently leads to a limited number of instances available for training effective and meaningful machine learning models. To address this issue and achieve adequate training data, reduce data disparity between cell lines, and yield robust synergy prediction results, data augmentation for the AZ-DREAM Challenges dataset was carried out. Each entry in the synergy dataset comprises a drug pair, a cancer cell line targeted by that pair, and a class label indicating whether these drugs exhibit synergy or antagonism when applied to that specific cell line. To unbiasedly scale up the dataset, new instances were generated by replacing molecules in drug pairs with chemically and pharmacologically similar molecules, under the assumption that similar molecules have similar therapeutic effects. Figure 3 shows that for one compound (drug *B*) in the original pair *A*:*B*, a set of candidates are selected from the STITCH database [44] (drugs *C*, *D*, and *E*). Subsequently, these molecules undergo evaluation through the DACS filter, which evaluates both the chemical structure and the drug action similarities [45]. This process ensures that the pharmacological profiles of the drugs chosen for augmentation (drugs *C* and *E*) closely resemble those of their parent molecule (drug *B*). Finally, class labels of the augmented instances *A*:*C* and *A*:*E* are directly transferred from the original drug combination *A*:*B*. This procedure is then repeated for the other compound in the pair (drug *A*) to create additional synthetic instances. Through data augmentation, the dataset for synergy classification was expanded from 3272 drug pairs, containing 76.6% synergistic and 23.4% antagonistic combinations, to as many as 2,315,325 instances, 79.9% of which are synergistic and 20.1% are antagonistic. Therefore, the ratio of synergy to antagonism is similar for the original and augmented datasets.

### 3.5. SynerGNet to Predict Drug Synergy

**Figure 4:**
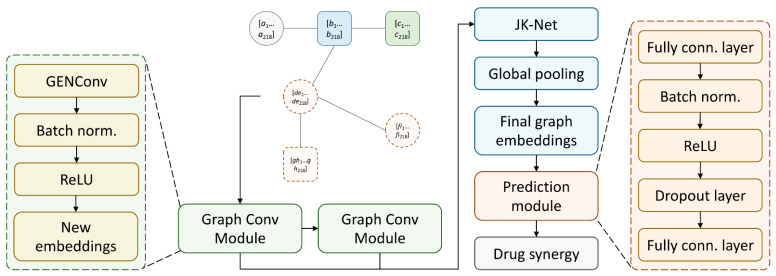
SynerGNet architecture. The input to SynerGNet is a reduced graph for a pair of drugs and a target cancer cell line. The graph is processed through a cascade of two graph convolutional modules propagating node information. At the end of each convolutional module, an updated new embedding is generated for each node in the graph. Embeddings generated by two graph convolutional modules are aggregated by the jumping knowledge network (JK-Net). A global pooling mechanism is used to extract graph-level features based on the node level features. In the final step, the graph embeddings are input into the prediction module, which predicts whether the paired drugs exhibit synergy or antagonism against the cancer cell line.

We developed a GNN model, SynerGNet, that can effectively capture the complexities of cancer cellular processes and the biochemical reactions of anticancer drugs, aiming to discern the patterns within the synergy data. The architecture of SynerGNet is presented in Figure 4. The input to the model is the graph representation (after reduction) of each synergy instance that consists of a pair of anticancer drugs and a target cancer cell line. The graph-structured data are first processed through a cascade of two graph convolutional modules. Each graph convolutional module is composed of a GENConv layer [52], followed by batch normalization and a rectified linear unit (ReLU). The GENConv facilitates deep learning on graph data and supports multiple aggregation functions for effective adaptation to diverse graph structures. Batch normalization normalizes features within a batch to a standard distribution. It helps alleviate a situation where the distribution of values among internal nodes undergoes significant changes during training, potentially hindering the learning process. This, in turn, promotes a more stable and expedited training process.

The ReLU layer introduces non-linearity to the model, enhancing its capacity to capture complex patterns. The structure of the graph convolutional module facilitates the propagation of node-level information in each graph. Embeddings from both convolutional modules are combined by the jumping knowledge network (JK-Net) layer [53], which aggregates feature representations from various network depths, thereby enriching the model’s ability to learn both local and non-local graph features. As drug synergy is a graph-level classification, a global pooling mechanism is used to extract the graph-level features to construct the final embeddings. The maximum and average values across nodes are extracted as features of the entire graph. Next, a prediction module is employed to convert the graph-level embeddings into the final representation that can be used for classification. This module comprises two fully connected layers with batch normalization, ReLU, and a dropout layer in between to improve the regularization of the model and, therefore, reduce overfitting. The graph-level embeddings are passed to this prediction module for the final prediction of the synergistic effect of two anticancer drugs against a cancer cell line.

### 3.6. Performance of Drug Synergy Predictors

**Figure 5:**
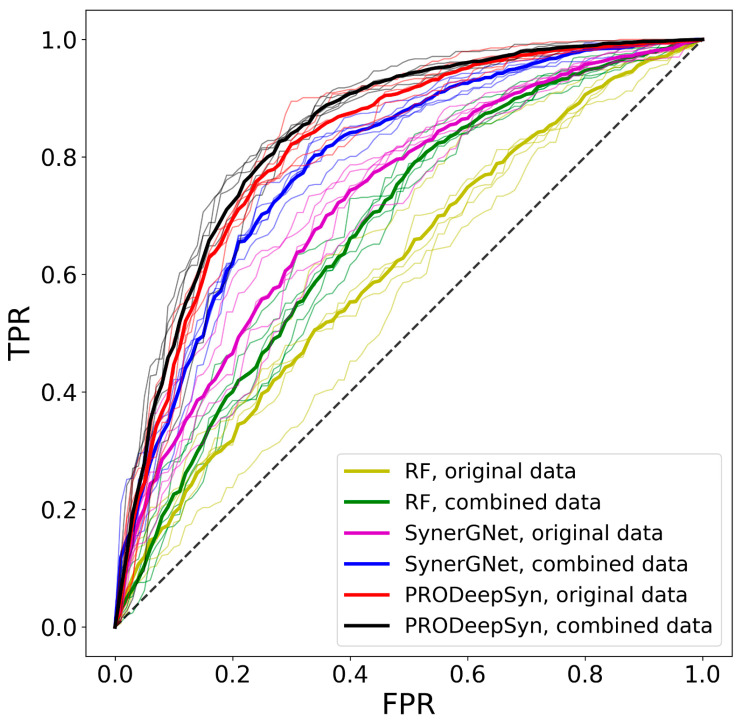
Performance of machine learning models to predict drug synergy. Receiver operating characteristic plots for random forest and SynerGNet, trained on the original AZ-DREAM Challenges data and the combined set of original and augmented data. Thick curves show the mean performance averaged over individual folds represented by thin curves. TPR—true positive rate, FPR—false positive rate.

The accuracy of RF, SynerGNet, and PRODeepSyn [54] are evaluated through a comprehensive analysis designed to understand the impact of including the augmented data in model training. For each classifier, a 5-fold cross-validation is conducted where the model is first trained solely on the original dataset and then on the combined set of original and augmented data. Note that the augmented data are only used to train machine learning models, which are then validated against the original AZ-DREAM Challenges instances excluded from training. The performance of RF, SynerGNet, and PRODeepSyn are reported in Table 2, with the corresponding ROC plots shown in Figure 5. It is evident that both classifiers perform better when the augmented data are incorporated during model training. Specifically, training RF against the combined original and augmented data results in a 6.6% increase in the area under the ROC curve (AUC), a 2.7% increase in the balanced accuracy (BAC), and a 10.1% improvement in the Matthews correlation coefficient (MCC) compared to training on the original data alone. Even higher improvements are noted for SynerGNet with the AUC increased by 6.9% and the BAC by 5.8%, the false positive rate (FPR) decreased by 7.3%, and a gain of 11.0% in the MCC when the model is trained on the combined versus the original data. These results clearly demonstrate that integrating augmented data during training enhances the ability of machine learning to more accurately differentiate between synergistic and antagonistic drug combinations. Moreover, this enhanced performance is observed across different tissue types (Table 3).

Another important observation is that SynerGNet consistently outperforms RF, according to all metrics. Notably, SynerGNet, even when trained solely on the original dataset, surpasses RF trained on the combined set of original and augmented data. Further, Table 2 shows that the ΔBAC, defined as the difference between training and testing BAC, for SynerGNet is an order of magnitude smaller than that for RF. A smaller ΔBAC indicates that SynerGNet has a better generalization capability, viz. the performance of the model is more stable for both seen and unseen data. This reduces overfitting by allowing the model to learn patterns generalizable to new data rather than memorizing the training dataset.

PRODeepSyn demonstrates a performance comparable to, or slightly better than, SynerGNet in the random split 5-fold cross-validation. The use of molecular fingerprints as features in PRODeepSyn contributes to this result, as the random splitting of instances into folds leads to similar drugs being assigned to different validation folds. Consequently, PRODeepSyn can achieve a high performance relatively easier by memorizing certain features compared to SynerGNet, which does not rely on molecular fingerprints. It is worth noting that the benchmarking calculations show more substantial improvements for SynerGNet when using augmented data compared to PRODeepSyn. This disparity can be attributed to the unique characteristics of SynerGNet, particularly its capacity to leverage augmented data effectively, a capability not shared by PRODeepSyn. In practical applications, where a consistent performance on new data is crucial, SynerGNet is anticipated to be particularly valuable. Despite the benchmarking differences, the expectation is that SynerGNet will demonstrate reliability and robustness in real-world scenarios, further enhancing its utility for applications beyond the benchmarking context.

### 3.7. Independent Validation of SynerGNet

To evaluate the capability of SynerGNet and PRODeepSyn to generalize to unseen data, both classifiers were applied to an independent validation dataset, DrugCombDB [49]. This dataset comprises 55 instances, including 6 synergistic and 49 antagonistic drug combinations, involving molecules with a low chemical similarity to those in the AZ-DREAM Challenges dataset. As in previous benchmarks, we employed models trained on both the original AZ-DREAM Challenges dataset and the combined set of original and augmented data. This choice was made to specifically investigate the enhancements in model generalizability resulting from the inclusion of augmented data during the training process. Table 4 indicates that the SynerGNet trained solely on the original data yields a modest predictive performance on the validation set, with metrics such as the AUC at 0.595 and the BAC at 0.485, for instance. On the other hand, SynerGNet trained on the combined set of original and augmented data exhibits substantial improvements, with the AUC increased to 0.748 and the BAC increased to 0.633. These enhancements indicate an improved overall capability of the model to accurately classify drug interactions within the validation conditions. Notably, the precision (PPV) increased to 0.143, and the MCC increased to 0.195. It is worth noting, however, that the higher FPR of 0.735 might suggest a potential bias towards predicting drug synergy, possibly reflecting the composition of the training AZ-DREAM Challenges data.

On the flip side, the performance of PRODeepSyn with respect to unseen data not only falls significantly short of that of SynerGNet but also fails to show improvement when trained on the combined set of original and augmented data. It is important to note that the performance drop of PRODeepSyn against unseen data is much more pronounced compared to its performance in the random split 5-fold cross-validation. This disparity arises from the reliance of PRODeepSyn on molecular fingerprints as features, a contrast to SynerGNet. The chemical similarity between drugs in the AZ-DREAM Challenges data (used for training) and those in the DrugCombDB dataset (used for validation) is notably low, with a mean TC of only 0.22 ± 0.07. Therefore, unlike random split benchmarking calculations where PRODeepSyn can memorize drug chemical features, its performance against unseen data is severely impacted. This underscores a key limitation of PRODeepSyn to leverage chemical similarities when confronted with unfamiliar drug data. In contrast, the ability of SynerGNet to generalize to unseen data is a notable strength, highlighting its capability to navigate challenges associated with limited original data. Overall, these findings not only underscore the aptitude of SynerGNet for generalizing to unseen data but also emphasize the potential of data augmentation to enhance drug synergy prediction, particularly in scenarios where the availability of original data is constrained.

## 4. Discussion

Utilizing graph-structured data to model cancer-specific biological networks based on PPIs offers notable advantages over traditional approaches. This technique effectively captures the intricate and non-linear interactions inherent in biological systems, which are often oversimplified in Euclidean space. The proposed representation proved particularly beneficial for employing advanced computational techniques such as GNNs, enhancing our capacity to interpret complex biological interactions and improve predictions of drug responses. However, the use of full-sized graphs, characterized by a uniform graph topology and large size, can present challenges for GNN models. Essential for addressing this issue, knowledge-based graph reduction techniques aim to increase data diversity, reduce computational complexity, and enhance the efficiency and interpretability of the GNN learning process [55].

Data augmentation was another important technique in overcoming the limitations posed by sparse drug synergy data. Our augmentation strategy effectively increased the volume of training data while preserving the distribution and class ratio of the original dataset. This expansion was not just quantitative but also qualitatively enriching, as it introduced a wider variety of instances for the models to learn from. The augmented data proved instrumental in boosting the performance of both graph deep learning based SynerGNet and a traditional RF classifier. It emphasized the significance of having a rich and diverse dataset in machine learning, especially for improving the accuracy of class differentiation and maintaining a balance between sensitivity and specificity. This underscores the extensive advantages of incorporating data augmentation in predictive modeling.

SynerGNet was demonstrated to consistently outperform traditional machine learning in all performance metrics for predicting anticancer drug synergy, even when trained with the original AZ-DREAM Challenges dataset. Importantly, SynerGNet exhibited desirable generalization capabilities, as evidenced by the smaller disparity between training and testing balanced accuracy. These performance achievements suggest its advanced ability in learning the intricate interplay of cellular mechanisms and drug interactions, which traditional techniques might not be able to effectively capture. Additionally, SynerGNet is less prone to overfitting and more capable of learning generalizable patterns, which is an essential quality for practical applications. Indeed, the results of an independent validation conducted against DrugCombDB provide compelling evidence that SynerGNet maintains its high accuracy when applied to unseen data.

The proposed SynerGNet model exhibits several key advantages in predicting drug synergy. Firstly, it integrates diverse molecular and cellular features into PPI networks, effectively capturing complex and non-linear interactions within biological systems, a task that is often oversimplified in models based on Euclidean format data. Secondly, SynerGNet utilizes drug–protein association scores instead of SMILES representations as the drug feature, ensuring that the biological connectivity and relationships between molecules and proteins are learned rather than merely memorizing drug structures, enhancing the model generalization to real, unseen data. Thirdly, the incorporation of a strong regularization mechanism in SynerGNet enhances its robustness to overfitting, ensuring more reliable prediction outcomes. Lastly, the model’s ability to benefit from large-scale augmented data demonstrates its suitability for extensive training, leading to an improved prediction accuracy and a balanced approach to sensitivity and specificity. Collectively, these attributes underscore its innovative approach and potential in the field of drug discovery.

While SynerGNet demonstrates a range of advantageous properties, it is also important to acknowledge its limitations. Firstly, the model faces challenges with full-sized graphs, which can increase computational complexity and potentially reduce the efficiency and interpretability of the learning process. When employing full-sized PPI networks in graph data construction, a graph reduction process becomes essential to facilitate effective learning in SynerGNet. Secondly, exclusive reliance on drug–protein association scores as drug features might lead to a lower testing accuracy compared to models that incorporate SMILES representations. This is because SynerGNet is tasked with the more complex challenge of extracting underlying patterns without the structural information typically provided by SMILES. Lastly, the performance of SynerGNet is notably enhanced by data augmentation, indicating a potential limitation in scenarios where diverse and augmented datasets are not available.

## 5. Conclusions

The development and validation of SynerGNet mark a noteworthy advancement in leveraging GNNs for the prediction of drug synergy. Several key conclusions can be drawn from this achievement. SynerGNet represents an innovative application of graph neural networks. GNNs are designed to process and analyze graph-structured data, and by extending their utility to predict drug synergy within complex biological networks, SynerGNet demonstrates the adaptability and versatility of GNNs in the realm of precision medicine. One of the strengths of SynerGNet lies in its ability to effectively capture intricate interactions within cancer-specific networks. Cancer networks are inherently complex, involving numerous molecular entities and signaling pathways. By successfully navigating and comprehending these intricate interactions, SynerGNet provides a sophisticated approach to understanding the complexities of cancer biology. SynerGNet’s utilization of augmented drug synergy data underscores its capacity to enhance learning from an enriched dataset. Data augmentation techniques, such as incorporating additional information or artificially expanding the dataset, contribute to its ability to recognize and predict drug synergies with improved accuracy. This utilization of augmented data is crucial for enhancing the robustness and generalization capabilities of the model.

The successful development and validation of SynerGNet hold promising prospects for the field of precision oncology. Predicting drug synergy is pivotal in tailoring treatments to individual patients based on the specific molecular characteristics of their cancer. SynerGNet’s ability to navigate cancer-specific networks opens avenues for more targeted and personalized therapeutic interventions, aligning with the goals of precision medicine. Further, SynerGNet’s capabilities have implications for drug discovery by facilitating the identification of synergistic drug combinations. The model’s predictive accuracy and its understanding of intricate network interactions provide a valuable tool for researchers and pharmaceutical companies seeking to optimize drug combinations that demonstrate enhanced therapeutic effects. Finally, the successful development of SynerGNet establishes a solid foundation for future research in the intersection of graph-based methodologies, machine learning, and drug synergy prediction. Researchers may build upon the SynerGNet framework to further refine and expand its applications, contributing to ongoing advancements in the fields of oncology and drug discovery. In summary, SynerGNet represents a significant leap forward in harnessing the power of GNNs for predicting drug synergy within cancer-specific networks. Its achievements have broad implications for both precision oncology and drug discovery, offering a promising tool for unraveling the complexities of cancer biology and optimizing therapeutic strategies for improved patient outcomes.

## Figures and Tables

**Table 1:** Statistics for featured graphs. Two sets of graphs are considered, full-size and reduced graphs constructed through an edge contraction operation. All full-sized featured graphs have the same properties. Since the process of graph reduction introduces topological diversity, the individual properties of reduced featured graphs are reported as the mean ± standard deviation.

Graph Property	Full-Size Graphs	Reduced Graphs
Number of nodes	18,997	1372 ± 195
Number of edges	697,185	4204 ± 3083
Average node degree	73.40	5.78 ± 2.90
Graph density	0.00386	0.00408 ± 0.00135
Graph diameter	9	6
Ratio of druggable/non-druggable nodes	3.1	0.29 ± 0.15

**Table 2:** Performance of classifiers to predict drug synergistic effects. The 5-fold cross-validated performance of random forest (RF) is compared to that of SynerGNet and PRODeepSyn. Classifiers trained on the original AZ-DREAM Challenges data and the combined set of original and augmented data are evaluated against the original instances excluded from model training.

Classifier	Dataset	AUC	BAC	PPV	FPR	MCC	ΔBAC
RF	Original	0.612	0.572	0.800	0.635	0.142	0.222
	Combined	0.678	0.599	0.807	0.711	0.243	0.336
SynerGNet	Original	0.721	0.676	0.863	0.380	0.313	0.032
	Combined	0.790	0.734	0.892	0.307	0.423	0.075
PRODeepSyn	Original	0.819	0.724	0.927	0.156	0.379	0.066
	Combined	0.838	0.730	0.931	0.147	0.389	0.089

AUC—area under the receiver operating characteristic plot, BAC—balanced accuracy, PPV—precision, FPR—false positive rate, MCC—the Matthews correlation coefficient, ΔBAC—difference between training and testing BAC.

**Table 3:** Performance of SynerGNet on different tissues. The classifier is 5-fold cross-validated with training conducted against the combined original and augmented data, and then evaluated using cell lines belonging to a specific tissue in the original data.

Tissue	AUC	BAC	PPV	FPR	MCC
Breast	0.778	0.698	0.869	0.418	0.375
Digestive system	0.683	0.637	0.807	0.473	0.265
Excretory system	0.793	0.710	0.831	0.288	0.402
Respiratory system	0.768	0.653	0.908	0.593	0.297
Other	0.843	0.720	0.883	0.140	0.518

AUC—area under the receiver operating characteristic plot, BAC—balanced accuracy, PPV—precision, FPR—false positive rate, MCC—the Matthews correlation coefficient.

**Table 4:** Performance of SynerGNet and PRODeepSyn on the DrugCombDB dataset. The classifiers trained on the original AZ-DREAM Challenges data and the combined set of original and augmented data are evaluated against an independent validation set from DrugCombDB.

Classifier	Training Set	AUC	BAC	PPV	FPR	MCC
SynerGNet	Original	0.595	0.485	0.103	0.531	−0.019
	Combined	0.748	0.633	0.143	0.735	0.195
PRODeepSyn	Original	0.159	0.308	0.036	0.551	−0.239
	Combined	0.092	0.437	0.096	0.959	−0.172

AUC—area under the receiver operating characteristic plot, BAC—balanced accuracy, PPV—precision, FPR—false positive rate, MCC—the Matthews correlation coefficient.

## Data Availability

Code and data are available at https://github.com/MengLiu90/SynerGNet (accessed on 1 December 2023).

## Abbreviations

AI: artificial intelligence; AUC: area under the curve; AZ-DREAM: AstraZeneca dialog for reverse engineering assessments and methods; BAC: balanced accuracy; CCLE: Cancer Cell Line Encyclopedia; CGM: chemical-genetic interaction matrix; CNV: copy number variation; DACS: drug action/chemical similarity; DL: deep learning; DT: decision tree; DTF: deep tensor factorization; FM: factorization machines; FPR: false positive rate; GAECDS: graph autoencoder with convolution neural network for drug synergy prediction; GENConv: generalized graph convolution; GNN: graph neural network; GO: gene ontology; JK-Net: jumping knowledge network; LR: logistic regression; MCC: Matthews correlation coefficient; ML: machine learning; NB: naïve Bayes; PCA: principal component analysis; PPI: protein–protein interaction; PPV: precision; ReLU: rectified linear unit; RF: random forest; ROC: receiver operating characteristic; SMILES: simplified molecular-input line-entry system; SONAR: second order naïve Bayesian and random forest; SVM: support vector machines; TLMCS: two-layer multiple classifier system; XGBoost: extreme gradient boosting.
